# The evolving landscape of eyebrow reconstruction: from traditional flaps to minimally invasive techniques and regenerative medicine

**DOI:** 10.3389/fsurg.2026.1865502

**Published:** 2026-08-06

**Authors:** Renjun Zhang, Hongxiao Tu, Huan Xiang, Xiaojie Hu, Zheyan Chen

**Affiliations:** 1 Department of Ophthalmology, Wuyi First People’s Hospital, Wuyi, Zhejiang Province, China; 2 Department of Ophthalmology, Wenzhou Third Clinical Institute Affiliated to Wenzhou Medical University, The Third Affiliated Hospital of Shanghai University, Wenzhou People’s Hospital, The Wenzhou People’s Hospital Affiliated of Hangzhou Medical College, Wenzhou, China; 3 Department of Plastic and Reconstructive Surgery, Wenzhou Third Clinical Institute Affiliated to Wenzhou Medical University, The Third Affiliated Hospital of Shanghai University, Wenzhou People’s Hospital, The Wenzhou People’s Hospital Affiliated of Hangzhou Medical College, Wenzhou, China

**Keywords:** eyebrow reconstruction, facial reconstruction, flap transfer, hair transplantation, minimally invasive techniques, regenerative medicine, tissue engineering

## Abstract

Eyebrow defects or deformities significantly impact facial aesthetics and emotional expression, making eyebrow reconstruction a critical area in reconstructive surgery. This review systematically examines the evolution of eyebrow reconstruction techniques, focusing on the innovative transition from traditional reliance on flap transfers to modern minimally invasive approaches and regenerative medicine strategies. The article provides an in-depth analysis of the principles, indications, clinical outcomes, and limitations associated with various techniques. It further explores current technical challenges and future directions in the field, aiming to offer a comprehensive theoretical reference and cutting-edge perspective for clinical practice. The integration of tissue engineering, hair transplantation, and advanced reconstructive methods holds promise for improving functional and aesthetic results, addressing both congenital and acquired eyebrow deficiencies.

## 1 Introduction

The eyebrow is a pivotal component of facial aesthetics and expression, serving not only as a protective barrier for the eyes but also as a key element in defining individual identity and conveying emotion. Its unique morphology, position, and hair density collectively contribute to the characteristic appearance of the face (1). Defects in the eyebrow, which can arise from a diverse array of etiologies including congenital anomalies, trauma, burns, post-tumor resection, or iatrogenic causes, impose significant psychological and social burdens on affected individuals. Consequently, the primary objective of eyebrow reconstruction is to restore a natural-looking brow with appropriate hair direction, texture, and long-lasting results. As a narrative review, this article provides an in-depth analysis of the principles, indications, clinical outcomes, and the evolution of techniques for achieving this goal, which mirrors the broader trajectory of plastic and reconstructive surgery., shifting from foundational, albeit sometimes invasive, methods toward more refined and patient-centric approaches. For decades, the use of local and regional flaps has been the cornerstone of surgical restoration. These techniques, such as the superficial temporal artery island scalp flap, have demonstrated reliability and versatility, particularly in managing challenging cases like post-burn alopecia (2, 3). The free superficial temporal artery flap has also been reported as a promising option for total eyebrow reconstruction, offering excellent vascularity and hair growth (4). Similarly, other pedicled flaps, including the paramedian forehead flap (with techniques for medial brow reintegration), the retroauricular scalp graft, and various local flaps like the orbicularis oculi muscle pedicled flap or the hemi glabellar flap, provide surgeons with a robust toolkit tailored to specific defect characteristics and locations (5–8).

However, traditional flap-based reconstruction is not without significant limitations. These procedures often involve donor site morbidity, potential distortion of adjacent facial landmarks (such as the hairline or contralateral brow), and challenges in precisely replicating the natural, fine hair direction and density of the native eyebrow. The inherent bulk of some flaps can lead to aesthetic displeasure, and the surgical footprint may be considerable (9, 10). Furthermore, the success of these methods can be heavily dependent on surgeon experience and the specific vascular anatomy of the patient. As the field has progressed, a heightened emphasis on aesthetic precision, minimal invasiveness, and functional preservation has driven innovation. This is evident in the meticulous attention now paid to the “brow-eye continuum,” where harmonious relationships between the eyebrow, eyelid, and forehead are crucial for optimal outcomes in both reconstructive and functional surgeries, such as ptosis correction (11, 12). The pursuit of such harmony has catalyzed the exploration and integration of novel strategies that promise greater control, reduced trauma, and more natural-appearing results, setting the stage for the current era of innovation in eyebrow restoration.

This review systematically examines the evolution of eyebrow reconstruction techniques, focusing on the innovative transition from traditional reliance on flap transfers to modern minimally invasive approaches and regenerative medicine strategies. The article provides an in-depth analysis of the principles, indications, clinical outcomes, and limitations associated with various techniques, aiming to offer a comprehensive theoretical reference for clinical practice1. Traditional Foundation of Eyebrow Reconstruction: Flap Grafting Technique.

### 1.1 Common flap types and their anatomical basis

**Figure 1:**
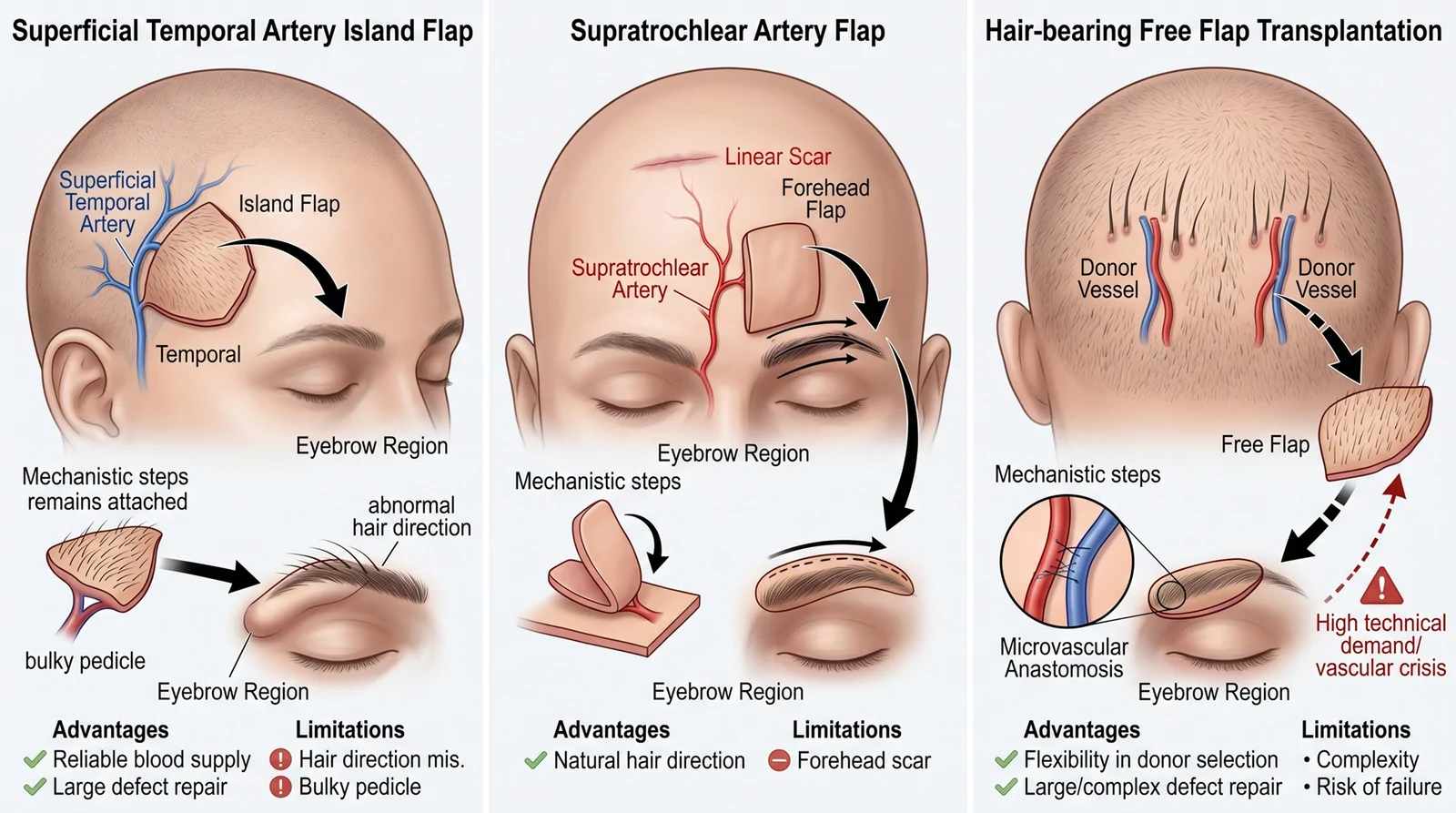
Traditional foundation of eyebrow reconstruction: flap grafting technique.

The superficial temporal artery island flap is a well-established workhorse for eyebrow reconstruction, particularly for large defects. This flap utilizes the superficial temporal vessels as its pedicle, allowing for the transfer of a hair-bearing scalp segment. The superficial temporal artery island scalp flap has been reported to be a reliable option for eyebrow reconstruction after burn alopecia (2, 3). This flap is especially valuable in challenging scenarios such as post-burn alopecia, where it can provide a substantial amount of tissue with reliable blood supply (2, 13). Preoperative planning with tools like CT angiography can further enhance safety by precisely mapping the superficial temporal artery and its often variable accompanying venous anatomy, allowing for a more accurate flap design (14). However, a significant limitation of this flap is the mismatch in hair characteristics; scalp hair transferred to the eyebrow region often differs in growth direction, texture, and density from natural eyebrow hair, which can result in a less natural appearance. Furthermore, the pedicle can sometimes lead to bulkiness at the donor or recipient site. The paramedian forehead flap, based on the supratrochlear or supraorbital vessels, is another cornerstone technique, primarily celebrated in nasal reconstruction. While not a first-line choice for isolated eyebrow defects, its use for large nasal defects can inadvertently disrupt the medial brow. Specific surgical techniques have thus been developed to reintegrate the medial eyebrow during the pedicle division stage of the forehead flap procedure, aiming to restore brow continuity and symmetry (5). This highlights the intricate relationship between forehead flap surgery and eyebrow aesthetics. For partial eyebrow defects, local flaps from the ipsilateral or even the contralateral eyebrow region are often considered the optimal choice due to their superior tissue match. A case report demonstrated the use of an orbicularis oculi muscle pedicled skin island flap from the contralateral eyebrow for reconstructing a partial lateral defect; for this procedure, local flaps from the ipsilateral remaining eyebrow have been noted to provide tissue with highly similar characteristics to the recipient site (7). Other local flap designs, such as the A-to-T advancement flap or T-plasty, have been applied in eyebrow reconstruction (15). The double rhomboid flap represents another local option, useful for reconstructing combined defects involving the eyebrow and medial upper eyelid, as demonstrated in a canine model, though it carries risks of complications like lagophthalmos (16). For the most complex and extensive defects, such as those involving large portions of the forehead and eyebrow, more sophisticated solutions are required. These can include the combination of a pedicled temporal hairline flap with a free forearm flap to provide both hair-bearing tissue for the eyebrow and ample coverage for the forehead (17), or the use of bilateral forehead rotation flaps to maintain symmetry while closing large central defects (18). The temporoparietal fascial flap (TPF), though thin and pliable, is less commonly a primary choice for eyebrow reconstruction itself but is a versatile tool in adjacent facial reconstruction; its harvest, especially in its composite form including scalp skin, is notably associated with donor site alopecia (19) (Figure 1).

### 1.2 Advantages and inherent limitations of traditional flap techniques

Traditional flap techniques for eyebrow reconstruction offer the fundamental advantage of providing a substantial, vascularized block of tissue in a single surgical stage. This is particularly critical for large or total eyebrow defects where simpler methods like grafting are insufficient. Flaps such as the superficial temporal artery island flap can deliver a reliable reconstruction in scenarios where follicular unit transplantation is not feasible (3). Their ability to bring their own blood supply makes them robust choices for compromised wound beds, such as those following burns or radiotherapy (2, 20). The stability of the result, once the flap has healed, is another key strength, providing patients with a permanent solution. However, these techniques are accompanied by significant and inherent limitations. The most conspicuous is the inevitable donor site morbidity. Harvesting a scalp flap leaves a linear scar and a permanent area of alopecia on the scalp (19), while using a forehead flap leaves a scar on the forehead, which can be a significant cosmetic concern (5). Even expanded forehead flaps, used primarily for nasal reconstruction, frequently result in noticeable bilateral eyebrow asymmetry and frontal deformation, issues that are acknowledged by a majority of patients despite overall satisfaction with the nasal outcome (21). Aesthetically, the mismatch between transferred scalp hair and natural eyebrow hair is a profound drawback. The hair direction, growth pattern, density, and texture often do not mimic the delicate, flat-lying hairs of a natural eyebrow, leading to results that can appear unnaturally coarse, bushy, or “hair-plug-like”. Sensory recovery in the transferred flap is often incomplete, leaving the reconstructed eyebrow with diminished or altered sensation. Furthermore, the transferred hair follicles may not thrive optimally in their new location; they can exhibit periodic shedding or poor long-term growth, compromising the final density. The surgical complexity of some flaps, particularly free flaps requiring microvascular anastomosis, introduces risks such as vascular compromise (vascular crisis), total flap loss, and demands a high level of surgical skill. While local flaps from the ipsilateral remaining eyebrow region itself offer better tissue match (7), they are limited by available donor tissue and are not suitable for large or total defects. In summary, while traditional flaps solve the fundamental problem of covering a defect with viable tissue, they often do so at the cost of suboptimal aesthetic refinement, donor site sacrifice, and potential functional compromises, highlighting the ongoing need for more sophisticated solutions in eyebrow reconstruction.

## 2 The infiltration of minimally invasive concepts: innovation of hair transplantation technology in eyebrow reconstruction

### 2.1 Evolution of single hair follicle unit transplantation techniques

Follicular unit extraction (FUE) has solidified its position as the dominant minimally invasive technique for eyebrow reconstruction, primarily due to its ability to avoid the linear scar associated with older strip harvest methods. Long follicular unit extraction (FUE) technique has been applied in eyebrow reconstruction with favorable outcomes (22). The atraumatic nature of this extraction is particularly emphasized in the context of cicatricial eyebrow loss, where careful handling is paramount to optimize graft survival in scarred recipient beds (23). The evolution of FUE has seen the development of specialized methods like long hair FUE, which allows surgeons to visualize the natural curl and direction of the hair during extraction and placement, a critical factor for achieving a natural-looking eyebrow where matching hair characteristics is essential (22, 24). Furthermore, the concept of “fine” FUE has been described, utilizing even more refined instrumentation to meticulously handle grafts, which has been associated with high hair survival rates and excellent cosmetic outcomes in patients with cicatricial defects (25). The transplantation process itself transcends mere technical execution, demanding a high degree of artistic design. This involves a detailed analysis of the natural eyebrow's anatomical subunits—the head (medial), body, arch, and tail (lateral)—and the meticulous planning of hair implantation parameters. Surgeons must replicate the natural gradient of density of native eyebrows to achieve favorable aesthetic outcomes. Equally critical is the precise control of the insertion angle of grafts and the direction of hair growth, which influences the aesthetic outcome of eyebrow transplantation (26). Failure to adhere to these natural patterns can result in sparse, disordered, or unnaturally dense eyebrows, leading to patient dissatisfaction (27). The advancement of transplantation is also heavily reliant on the refinement of implantation instruments. The use of ultra-fine needles, implanters, or pens allows for the creation of tiny, precise recipient sites. Refined FUE transplantation has been reported to achieve a postoperative hair survival rate of (84 ± 5)% in the treatment of scarred eyebrow defects (25). Single-follicle transplantation is applied in eyebrow reconstruction, and precise alignment of implantation angle and direction can improve aesthetic outcomes (26). The selection of the optimal donor hair source is another key aspect of technical evolution. While occipital scalp hair is a common source, its faster growth rate and thicker caliber compared to natural eyebrow hair can necessitate frequent trimming. Consequently, alternative donor sites with hair characteristics more closely resembling eyebrows have been explored. The frontal-temporal triangle area (FTTA) and periauricular area are noted for having slower-growing, finer hair, leading to more aesthetically pleasing and low-maintenance results in eyebrow restoration, as supported by comparative studies (28, 29). In challenging cases of scarred recipient beds, such as those from deep burns, the basic FUE technique is often integrated with preparatory treatments to improve the local tissue environment. Procedures like nanofat injection, microfat grafting, or fractional laser therapy (ablative or non-ablative) are performed prior to transplantation to enhance scar pliability, vascularity, and texture, which collectively contribute to significantly higher follicular survival rates and more satisfactory overall outcomes (30–32).

## 3 Adjunctive and future-oriented regenerative strategies

### 3.1 Clinically available adjunctive therapies

The survival of transplanted hair follicles, particularly in compromised recipient beds (e.g., scars secondary to burns or trauma), can be significantly improved through preconditioning of the target area. These adjunctive approaches harness regenerative principles to augment local vascularity, skin texture, and tissue pliability.

Key clinically available adjunctive strategies include:

Platelet-Rich Plasma (PRP): PRP, derived from autologous blood, is enriched with growth factors such as platelet-derived growth factor (PDGF), transforming growth factor-beta (TGF-*β*), and vascular endothelial growth factor (VEGF). Its injection into the recipient area prior to or immediately after follicular unit extraction (FUE) is intended to stimulate angiogenesis and establish a more favorable microenvironment for graft survival. Evidence from a prospective case series and a retrospective comparative study suggests that combining PRP with FUE in cicatricial alopecia improves clinical outcomes (33, 34).
Microfat and Nanofat Grafting: Autologous fat grafting represents a versatile tool. Microfat provides structural support and volume, whereas nanofat—rich in adipose-derived stem cells (ADSCs) and growth factors—is processed into a liquid form. Injection of nanofat into scarred recipient beds prior to FUE has been demonstrated to significantly improve graft survival rates and overall aesthetic outcomes in burn patients.
Ablative and Non-Ablative Fractional Lasers: Fractional lasers, such as the carbon dioxide (CO₂) laser, resurface skin by creating microcolumns of thermal injury, thereby stimulating collagen remodeling, increasing dermal pliability, and promoting neovascularization. A prospective comparative study and a retrospective analysis reported that combining fractional CO₂ laser therapy with FUE for post-traumatic eyebrow scars resulted in significantly higher follicular survival rates and more defined eyebrow shapes compared to FUE alone (35, 36). Similarly, a combined regimen of non-ablative fractional laser and microfat grafting before FUE enhanced mean graft survival rates to 85%.Beyond CO₂ lasers, the erbium-doped yttrium aluminum garnet (Er:YAG) laser represents another important ablative fractional platform. Compared to the CO₂ laser (wavelength 10,600 nm), the Er:YAG laser (wavelength 2,940 nm) has an absorption coefficient for water that is approximately 10–15 times higher, resulting in a narrower zone of thermal necrosis and less collateral thermal coagulation to surrounding tissues. This characteristic theoretically makes the Er:YAG laser more suitable for delicate periorbital and eyebrow regions where precision and minimal thermal damage are paramount. Although the retrieved literature lacks direct clinical studies of Er:YAG laser preconditioning for eyebrow FUE, its physical properties suggest potential utility in improving scar texture and promoting collagen remodeling while potentially reducing the risk of thermal injury to hair follicles. Future research should directly compare the efficacy of CO₂ and Er:YAG lasers for recipient-site preparation in eyebrow reconstruction to determine the optimal laser platform. Non-ablative fractional lasers, such as the 1,550 nm erbium-doped fiber laser, represent a less invasive alternative. These devices create microscopic thermal zones (MTZs) in the dermis without disrupting the epidermal barrier, thereby stimulating collagen remodeling and neovascularization with minimal downtime and reduced side effects such as erythema and crusting (37). The retrieved literature includes a study evaluating “combined non-ablative fractional laser and microfat graft treatment” as a preconditioning strategy prior to FUE, which demonstrated an average hair follicle survival rate of up to 85%. This evidence suggests that for patients with mild to moderate scarring or those prioritizing rapid recovery, non-ablative fractional lasers offer a safe and effective option. However, for severe hypertrophic or contractile scars, their penetration depth and remodeling capacity may be inferior to ablative lasers, necessitating individualized clinical decision-making based on scar severity.

These adjunctive therapies constitute a safe and effective strategy to expand the indications of FUE into more challenging, scarred recipient beds. Their primary role is to precondition the local biological environment to better accommodate the transplanted follicular units.

### 3.2 Preclinical tissue engineering and future directions (not Yet clinically available)

The ultimate objective of regenerative medicine in this domain is the *de novo* generation of hair follicles, thereby circumventing the inherent limitations of donor supply. This goal is pursued through two primary lines of investigation: cell-based therapies and tissue engineering.

#### 3.2.1 A. Cell-Based Therapies

Research efforts have concentrated on isolating and directing the differentiation of hair follicle stem cells (HFSCs), particularly those residing in the bulge region, with the aim of constructing functional follicular units. These stem cells, characterized by expression of markers such as CD34, K15, Lgr5, and Lgr6, possess substantial self-renewal capacity and multipotency (38–40).

Animal studies have provided robust proof-of-concept. Foundational work demonstrated that skin-derived precursors (SKPs), a type of mesenchymal stem cell, when seeded in a collagen-glycosaminoglycan matrix together with epidermal stem cells, could reconstitute skin with hair follicle formation in nude mice (41). This principle was further advanced using injectable fibrin hydrogels co-embedded with SKPs to induce *de novo* hair genesis in mice (42).

More recently, sophisticated three-dimensional (3D) culture systems have been developed. Hair follicle organoids (HFOs) derived from human induced pluripotent stem cell (iPSC)-derived ectodermal precursor cells can generate hair follicles both *in vitro* and *in vivo* (43).The advent of iPSC technology offers a revolutionary, potentially unlimited source of autologous folliculogenic cells, thereby bypassing the need for skin harvesting (44, 45).

#### 3.2.2 B. Scaffold-Based Tissue Engineering

Biodegradable scaffolds, fabricated from materials such as collagen, hyaluronic acid, and fibrin, serve as temporary three-dimensional structures that act as cell carriers. The selection of scaffold material is critical, as it must provide appropriate mechanical support and biochemical cues to guide organized hair follicle morphogenesis. For instance, fibrin hydrogels have been shown to preserve the viability and stemness of SKPs while enhancing the expression of hair-inductive genes (46).

The synergistic incorporation of growth factors into scaffolds represents a key strategy. Injectable platelet-rich fibrin (i-PRF), a biomaterial rich in growth factors, promotes human dermal papilla cell (hDPC) proliferation and trichogenic inductivity (47). Furthermore, the combination of mesenchymal stem cell (MSC) secretome—a cocktail of bioactive factors—with Wnt10b protein within a 3D-printed gelatin methacryloyl (GelMA) hydrogel scaffold achieved a high rate of hair follicle formation after implantation into nude mice (48).

#### 3.2.3 C. Gene Editing (Conceptual Future)

Parallel to these approaches, gene editing technologies such as CRISPR-Cas9 present a transformative—albeit distant—possibility. This technology could theoretically be employed to precisely modify genes in transplanted cells to regulate hair growth cycle duration, shaft diameter, or pigmentation, thereby enabling ultimate customization (49–51).

#### 3.2.4 Critical disclaimer and future challenges

Despite these promising advances, substantial obstacles must be overcome before stable and controllable clinical applications for eyebrow reconstruction become feasible. These technologies—including hair follicle organoids, iPSC-derived cells, and advanced scaffold constructs—are currently confined to *in vitro* and animal models and have not yet been translated to human patients for eyebrow reconstruction. Key challenges include:

Controlling hair cycle and orientation: Achieving proper hair follicle orientation and a synchronized, durable growth cycle remains technically demanding.
Scalability and reproducibility: Reliable, standardized production of off-the-shelf constructs is a major manufacturing hurdle.
Immunogenicity and safety: Ensuring the long-term safety of cell-based or gene-edited products is paramount.
Regulatory pathway: Navigating the complex regulatory approvals required for cell and gene therapies is a protracted and rigorous process.

**Figure 2:**
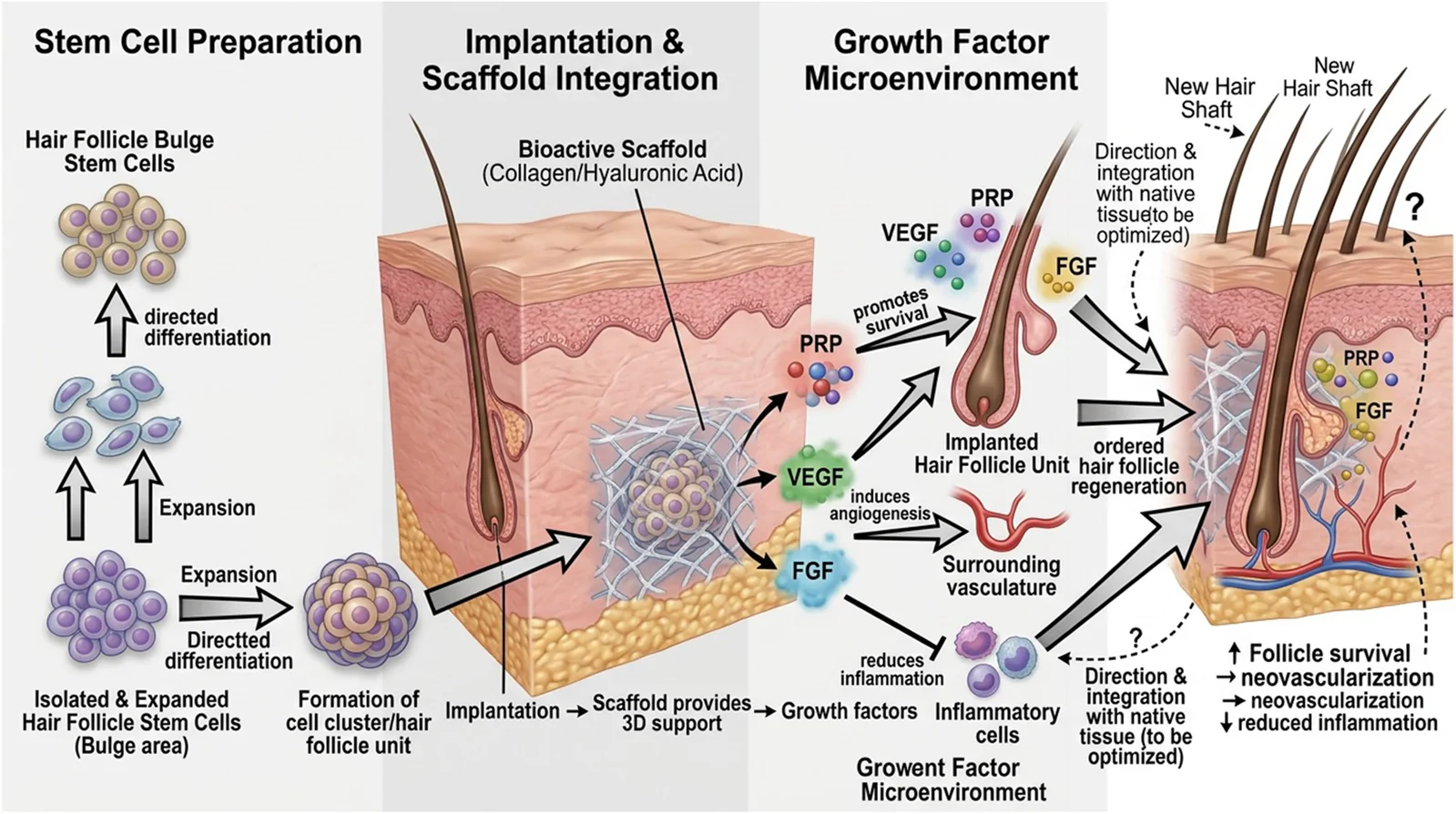
Introduction to regenerative medicine: tissue engineering and bioactive strategies.

The translation from successful animal models to reliable clinical therapy for eyebrow reconstruction is therefore complex. While these regenerative strategies represent the most promising frontier for achieving truly scarless and donor-independent restoration, they are not yet a clinical reality (Figure 2; Table 1).

**Table 1:** Current status of regenerative approaches for eyebrow reconstruction.

Approach	Current status	Clinical availability for Eyebrow Reconstruction
Platelet-Rich Plasma (PRP)	Clinical adjunct	Available to improve graft survival
Microfat/Nanofat grafting	Clinical adjunct	Available for improving scarred recipient beds
Fractional laser (CO₂, etc.)	Clinical adjunct	Available for scar remodeling before transplant
3D bio-engineered scaffolds + cells	Preclinical	Active research; promising animal models; no human data for eyebrow reconstruction
Induced pluripotent stem cell (iPSC)-derived follicles	Preclinical/Conceptual	Early proof-of-concept *in vitro*; significant scientific and regulatory hurdles remain
CRISPR gene editing for hair traits	Conceptual	Theoretical application; major ethical and safety concerns

## 4 Comprehensive assessment: comparison of indications and efficacy across different technical approaches

### 4.1 Surgical procedure selection strategy based on defect etiology and scope

**Figure 3:**
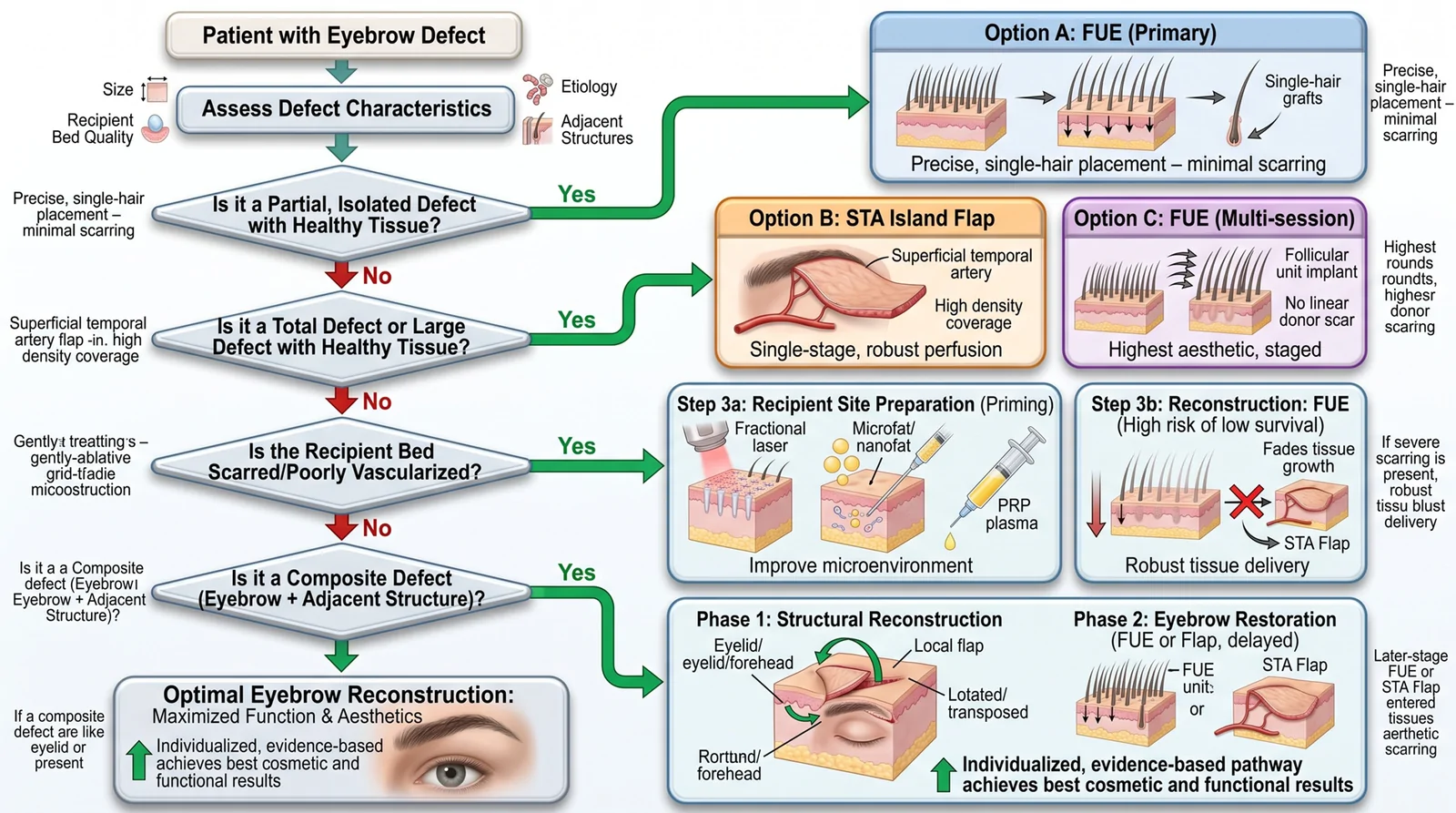
Clinical algorithm for eyebrow reconstruction technique selection.

The strategic selection of an eyebrow reconstruction technique is fundamentally dictated by the specific etiology and extent of the defect, requiring a nuanced approach that balances aesthetic outcome with the least invasive means possible. For localized, partial hair loss, often resulting from failed cosmetic procedures or localized scarring, follicular unit extraction (FUE) represents the preferred first-line approach due to its minimally invasive nature and capacity for producing natural-appearing results. This is particularly relevant for conditions such as scarring from prior burns or trauma where the recipient bed may be compromised; studies have demonstrated that hair transplantation using FUE can achieve high graft survival rates in children with cicatricial alopecia, improving appearance with low surgical risk (52). Furthermore, for post-burn alopecia in adults, FUE has emerged as a versatile procedure that can restore a natural anatomical profile, even on skin grafted areas, facilitating social reintegration and improving quality of life (41). However, it is critical to recognize that the biological properties of scar tissue can reduce graft survival. To mitigate this, a preparatory regimen combining non-ablative fractional laser and microfat injection before hair transplantation has been shown to significantly enhance graft survival rates to a mean of 85%, offering a promising solution for patients with poor recipient sites (32). In cases of complete eyebrow loss where the local scalp tissue is healthy and adequate, the superficial temporal artery (STA) island scalp flap remains a highly reliable and time-tested option. This technique is particularly advantageous for male patients or those requiring a high density of hair, providing a one-stage solution with a robust blood supply. The enduring utility of this flap is well-documented, especially in deep burn injuries of the face where it provides a versatile and effective means of reconstruction (2). Contemporary refinements, such as the use of preoperative CT angiography to map the course of the parietal branches of the STA and superficial temporal vein, have enhanced the safety and precision of this flap, ensuring vascular compromise is minimized (14). The role of the STA flap is so fundamental that even in advanced techniques, such as the free flap, the vessels are used; the first documented use of a free superficial temporal artery flap for total eyebrow reconstruction demonstrates the enduring principle of utilizing hair-bearing scalp with a dependable vascular axis (4). A more challenging scenario is the composite defect, where eyebrow loss is concomitant with a deficiency of adjacent structures like the eyelid or forehead skin. In these instances, reconstructive efforts must prioritize the restoration of the soft tissue contour and structural framework before considering hair replacement. For example, large defects around the eyebrow can be successfully repaired using an ultrathin supratrochlear artery cutaneous branch flap, which avoids facial distortion and provides good aesthetic outcomes in a single stage (53). When the defect involves the upper eyelid and is full-thickness, a complex approach may be necessary, such as combining a nasal septal chondromucosal graft for internal lining and cartilage support with a forehead transposition flap designed to include the eyebrow line, thereby reconstructing the eyelash simultaneously (54). Even more extensive defects involving large portions of the forehead and upper eyelid may necessitate the combination of two flaps, such as a pedicled temporal hairline flap for the eyebrow and a free forearm flap for the forehead skin, demonstrating a staged, strategic approach to complex composite tissue loss (17). Ultimately, the choice of technique is a hierarchical decision guided by the defect's three-dimensional nature, where simple partial defects are addressed with micrografting, complete eyebrow loss with vascularized scalp flaps, and composite defects with multi-staged, often flap-based, restorations of form and function (Figure 3).

### 4.2 Multidimensional analysis of aesthetic effect and patient satisfaction

A comprehensive assessment of outcomes in eyebrow reconstruction extends far beyond mere hair survival, necessitating a multi-dimensional evaluation of aesthetic harmony and patient-reported satisfaction that encompasses symmetry, naturalness, scar concealment, and dynamic integration with facial expressions. The aesthetic ideal for a reconstructed eyebrow includes precise symmetry of shape and position, a natural direction and angle of hair growth, a gradient of hair density from medial to lateral, and the ability to move synchronously with the contralateral brow during expressions of surprise, concern, or happiness. While the established “kite flap” technique for mild-to-moderate defects has demonstrated excellent results, with 66.67% of patients reporting being “very satisfied” and significant improvements in Face-Q scores for appearance and psychosocial function (55), the gold standard for achieving the most natural result in scar-related alopecia is often a standardized, microsurgical single-hair follicle transplantation technique combined with follicular unit extraction. This meticulous method can yield an average graft survival rate of 85% and a high patient satisfaction rate, with 95% of patients reporting high satisfaction with density and natural appearance after a first stage (56). This is a significant improvement over the 75% survival rate historically reported for similar procedures, underscoring how technical refinement directly enhances aesthetic outcomes. The concept of the “brow-eye continuum” is critical; ptosis correction surgeries, such as the frontalis muscle advancement technique, must be carefully planned to avoid disrupting this harmony, as postoperative eyebrow height can decrease, affecting the overall aesthetic result (11). This interplay is further highlighted in techniques like the orbicularis resection blepharoplasty, which can slightly elevate the eyebrow position, a change that may be desirable for some patients but unwanted for others (57). Long-term satisfaction studies reveal a distinct divergence in patient priorities between minimally invasive and traditional approaches. Patients who undergo micrografting (e.g., FUE) consistently report higher scores for “naturalness” and psychological acceptance, likely because the results are less prone to the “unnatural” look of a thick, depilated flap. However, they often require multiple sessions to achieve the desired density, which can be a source of frustration. Conversely, patients receiving traditional pedicled flaps, such as the superficial temporal artery island flap, have been reported to generally report high satisfaction with reconstruction outcomes (3). Notably, even advanced flap techniques, such as the “scalping forehead flap” or paramedian forehead flap used for large nasal defects, necessitate a secondary procedure to restore medial brow continuity, a step that is crucial for final patient satisfaction and facial symmetry (5). The psychological and social impact of these outcomes cannot be overstated. For patients with severe deformities, such as those from burns, even a modest improvement can dramatically enhance psychosocial function and quality of life. In cases of severe post-burn alopecia with facial distortion, sequential FUE sessions have been shown to facilitate proper social reintegration and improvement of overall quality of life (41). Meanwhile, patients undergoing corrective procedures for facial nerve dysfunction, such as chemodenervation and surgery, place the highest value on “care experience,” smiling, facial symmetry, and access to surgical treatments, emphasizing that while aesthetic results are paramount, the patient's journey and feeling of being understood are equally critical (58). Even more subtle outcomes, such as the impact of skin excision shape during blepharoplasty on pretarsal show homogeneity, demonstrate that patient-reported outcomes (FACE-Q scores) can be high with different techniques, yet objective measures like lateral brow descent can differ, indicating that surgeons must be attuned to both objective metrics and subjective patient satisfaction (59). Ultimately, the most successful eyebrow reconstruction strategy is one that aligns the technical possibilities with the patient's specific aesthetic goals, psychological needs, and tolerance for staged procedures, ensuring that the final result not only restores hair but also reintegrates the patient's facial identity (Table 2).

**Table 2:** Comparison of Key eyebrow reconstruction techniques.

Criteria	Traditional flaps (e.g., STA Island Flap)	Follicular unit extraction (FUE)	Regenerative medicine (Current/Future)
Primary Indications	Large, total, or composite defects; scarred beds with poor vascularity	Partial to total non-scarring alopecia; selected scar defects requiring refinement	Primarily experimental; potential for donor-independent, scarless total reconstruction
Key Advantages	Single-stage reconstruction; robust blood supply; provides bulk and tissue	Minimally invasive; no linear donor scar; precise control over hair direction/density; high aesthetic potential	Potential unlimited donor source; aim for truly regenerated, natural hair; eliminates donor site morbidity
Primary Limitations	Donor site morbidity (scar, alopecia); mismatch in hair texture/direction; potential bulkiness	Multiple sessions may be required for density; limited by donor hair supply; relies on recipient bed quality	Highly experimental; challenges in controlling hair cycle, orientation, and pigmentation; scalability uncertain
Number of Stages	Typically 1 stage	Often 1–2 stages	Currently experimental; likely multi-stage
Aesthetic Outcome	Functional but often lacks fine detail; can appear “patch-like” or unnatural	High potential for natural, refined, undetectable reconstruction (with skill)	Theoretically indistinguishable from native brow; long-term stability unknown
Graft/Flap Survival	Very high (>95% flap survival)	Moderate-High (85%–95% graft survival in optimal conditions)	Currently low and unpredictable *in vivo*
Patient Satisfaction	Generally satisfied with defect closure; lower satisfaction with ultimate aesthetics	High satisfaction with naturalness; lower with need for multiple sessions	Not applicable at clinical level

## 5 Core challenges and bottlenecks currently faced

### 5.1 Standardization of technical operation and learning curve of doctors

The advancement of eyebrow reconstruction techniques, particularly the adoption of Follicular Unit Extraction (FUE), has introduced a critical challenge centered on the lack of standardized technical protocols and a steep learning curve for surgeons. While FUE offers the benefit of avoiding a linear donor scar, its application in the eyebrow region demands an exceptionally high level of artistic sensibility and microsurgical dexterity, making outcomes heavily dependent on individual practitioner experience. A retrospective analysis of poor eyebrow transplant outcomes revealed that 41% of dissatisfied patients exhibited inhomogeneous density and disordered growth direction, and 36% had sparse eyebrows, all of which are direct consequences of poor surgical planning and execution (27). These findings underscore that achieving a natural, aesthetically pleasing eyebrow is not merely a matter of follicle survival but requires meticulous attention to the native hair's geometric pattern. To address this variability, a nine-step standardized operating procedure has been proposed for scarring alopecia, which emphasized the need for a consistent workflow to optimize outcomes (56). This protocol reportedly achieved a high satisfaction rate of 95% and an average graft survival rate of 85%, significantly higher than previously reported figures, demonstrating that standardization can dramatically improve results (56). However, even with such protocols, the procedure remains technically demanding. The learning curve is further complicated by the need to customize the approach for each defect, as evidenced by the diverse range of techniques employed, from local flaps for partial defects to island flaps for total reconstruction (4, 7). The situation is further nuanced by gender-specific aesthetic ideals; for instance, a large-scale study of East Asians found that females prefer a smooth arched eyebrow, while males prefer a triangular straight shape (1). This requires surgeons to not only master the technical skill of implantation but also possess a deep understanding of ethnic and gender-specific facial aesthetics to tailor the procedure appropriately. Furthermore, the integration of regenerative medicine concepts from the laboratory to the clinic involves a long and arduous pathway. This includes navigating strict regulatory approvals, ethical reviews, and the establishment of standardized production processes for cell-based therapies or bioengineered constructs. The complexity of these requirements means that while such technologies hold great promise for “scarless” reconstruction, their widespread clinical application remains a distant reality, with current practice heavily reliant on the refined yet non-standardized, skills of the surgeon during the transplantation of natural hair follicles. Therefore, the field's main challenge is not the lack of effective techniques, but the need for robust, evidence-based standardization that can flatten the learning curve and democratize high-quality outcomes, a goal that remains elusive even as it is increasingly recognized as essential.

### 5.2 Survival rate and long-term stability of hair follicles

Even with the most refined transplantation techniques, achieving 100% survival of grafted hair follicles in eyebrow reconstruction is an elusive goal, and the long-term stability of the regenerated hair remains a significant concern. The survival and growth of transplanted follicles are threatened by multiple factors. Ischemia-reperfusion injury occurs when the follicle is removed from its high-oxygen environment in the donor area and then implanted into a relatively hypoxic recipient site, a process that can trigger cellular damage and death. The quality of the recipient bed is also paramount; scarred tissue, such as that from burns or trauma, often has poor blood supply, which can significantly compromise graft survival (2, 3). Clinical data from a large study of scarring alopecia showed that even with a refined standardized technique, the average graft survival rate was approximately 85%, which, while a significant improvement, still represents a 15% loss of grafts (56). Other studies have noted low hair survival rates as a primary reason for patient dissatisfaction post-transplantation (27). Beyond initial survival, the long-term stability and growth cycle of the transplanted hair are critical. After transplantation, hair follicles typically enter a dormant telogen phase and shed within the first few weeks, a process that can be alarming for patients. The subsequent regrowth occurs in a new cycle, which may not perfectly mirror the native eyebrow's growth rhythm. There is evidence suggesting that the growth period (anagen) of transplanted scalp hair may remain longer than that of natural eyebrow hair, leading to a secondary issue of hair that grows too long and requires frequent trimming. Furthermore, the cyclical nature of hair growth means that periodic shedding can occur, necessitating long-term follow-up to monitor stability. While the cycle of transplanted natural hair is well-documented, the long-term cycle control of regenerate hair from emerging technologies is a completely unknown domain. For instance, if bioengineered hair follicles are implanted, their response to native signaling pathways, their potential for cyclical regeneration, and their long-term viability remain uncharacterized. The challenge is compounded by the influence of the recipient site microenvironment. For example, cases of burned skin may have altered vascularity and scarring that not only affect initial survival but may also affect the long-term health of the graft (2). Even in less hostile environments, the long-term survival of grafts can be impacted by factors like subclinical inflammation and the patient's own hormonal status. Although techniques such as platelet-rich plasma, microneedling, and fat grafting are being explored to improve graft survival and the condition of the recipient bed, their long-term impact on follicular stability is still under investigation (23). The ultimate goal is not only to transplant follicles that survive but to integrate them into a stable, self-renewing system that provides a permanent, natural-looking eyebrow, a goal that current techniques achieve with a significant degree of success, but not perfection.

### 5.3 Contradiction between supply area resource limitation and traceless demand

A fundamental limitation in current eyebrow reconstruction techniques, particularly those relying on follicular unit extraction (FUE), is the inherent conflict between the finite resources of the donor supply and the increasing patient demand for a completely scarless outcome. The standard of care involves harvesting hair follicles from the occipital scalp, which is considered the most “permanent” donor area. However, this approach is intrinsically limited for patients with insufficient donor reserves, such as those with advanced androgenetic alopecia (male or female pattern baldness). For these individuals, the occipital scalp may have a reduced density of viable follicles, making it impossible to harvest enough grafts to create a look of sufficient density. In such cases, alternative donor sites might be considered, such as the beard or chest hair, but these often have different characteristics in texture, color, and growth cycle compared to eyebrow hair, potentially leading to a less natural aesthetic (60). The conflict is further sharpened by the patient's desire for an “invisible” donor site. While FUE is marketed as “scarless,” the reality is that it creates thousands of tiny, circular scars in the donor area, which, when shaved down, can create a subtle but visible stippled pattern. This aesthetic trade-off is unacceptable for some patients, especially those who may have cosmetic concerns about their scalp. The drive to eliminate this donor-site morbidity has fueled the search for “donorless” solutions, primarily through the avenue of regenerative medicine, such as hair cloning or *de novo* follicle generation. However, as noted earlier, these technologies remain experimental and face immense scientific and regulatory hurdles before they can become a clinical reality. Therefore, clinicians are forced to navigate a paradox: the more a patient demands a totally scarless reconstruction, the more they may require a form of technology that does not yet exist. In many cases, especially for large defects, surgeons have had to resort to other techniques that do not avoid a donor scar but provide a more predictable outcome. For example, the use of a scalp island flap based on the superficial temporal artery provides a large, hair-bearing unit with a robust blood supply but involves a visible linear scar on the temporal scalp (2, 3). Similarly, a retroauricular scalp graft, while offering excellent hair texture match, creates a scar behind the ear (6). The tension between the ideal of “no scar” and the practical realities of finite donor supply remains a central challenge, pushing the field to innovate in both surgical technique (e.g., refining FUE to minimize scarring) and in the pursuit of truly donor-independent regenerative strategies.

## 6 Multidisciplinary integration and personalized precision medicine model

### 6.1 Multidisciplinary collaboration

The optimization of eyebrow reconstruction, from preoperative preparation to intraoperative execution and postoperative management, fundamentally relies on a well-coordinated multidisciplinary team approach. This collaborative model is essential for managing complex cases, such as severe composite facial defects resulting from trauma or oncologic resection, where expertise beyond a single surgical specialty is required. For instance, the successful management of a patient with a severe bear maul injury involving the forehead, eyebrow, eyelids, and cheek necessitated a coordinated effort from ophthalmology, otorhinolaryngology, and plastic and reconstructive surgery to achieve ocular salvage, functional restoration, and an acceptable cosmetic outcome (61). Similarly, the primary reconstruction of a traumatic hemifacial avulsion and degloving injury, which compromised the airway and involved the eyebrow, was effectively managed through a rapid multidisciplinary response (62). This team-based framework extends to the preoperative phase, where collaboration can facilitate comprehensive medical optimization. While the provided references do not specifically detail the use of preoperative medications like minoxidil to improve recipient site vascularity, the principle of integrated care is evident in cases requiring staged reconstruction. For example, a complex pediatric nasal and eyebrow reconstruction in an immunocompromised patient with recurrent HSV infection was safely executed over five stages, a feat made possible by careful vascular assessment and multidisciplinary collaboration to navigate the heightened perioperative risks (63). Postoperatively, the collaborative model supports adjunctive treatments to refine outcomes. Although not explicitly mentioned for eyebrow procedures in the given texts, the use of laser technology for scar management and pigmentation is a recognized adjunct in reconstructive surgery. A systematic review highlights the role of light and laser devices as promising therapeutic options for keratinization disorders like keratosis pilaris atrophicans faciei, which can affect cosmetic results (64). Furthermore, for challenging scenarios like hair transplantation into burn scar alopecia affecting the eyebrow, a combined approach utilizing non-ablative fractional laser and microfat grafting prior to follicular unit extraction has been shown to significantly improve graft survival rates and patient satisfaction, demonstrating the synergy between laser technology, regenerative techniques (fat grafting), and surgical hair restoration (32). This integrated strategy ensures that every phase of care—addressing vascularity, executing precise surgery, and managing scars or promoting graft growth—is handled by the appropriate specialist, thereby maximizing functional and aesthetic results for the patient.

### 6.2 Preoperative design and simulation based on digital technology

The integration of digital technology into the preoperative planning of eyebrow reconstruction represents a significant advancement toward achieving precise, patient-specific, and aesthetically congruent outcomes. While the provided references do not explicitly mention Computer-Aided Design (CAD) or virtual reality for eyebrow design, the underlying principle of precise planning and measurement is strongly supported. The use of cephalometric analysis, for instance, provides a quantitative, imaging-based method for assessing facial proportions and planning interventions. A prospective study utilized cephalometric measurements to quantitatively evaluate the efficacy of hyaluronic acid and poly-L-lactic acid injections for facial rejuvenation, specifically tracking changes in eyebrow peak and tail angles, and distances from the eyebrow to the orbital rim (65). This objective, data-driven approach to planning and assessing outcomes in periorbital and eyebrow aesthetics underscores the value of precise measurement, which forms the foundation for more advanced digital simulation. The concept of personalized, precise intervention is further highlighted in neuromodulator treatments. Research into increasing injection precision for frontal rhytids has explored the use of pre-injection high-frequency ultrasound imaging to identify individual anatomical landmarks, such as the “line of convergence” of the frontalis muscle, to avoid complications like eyebrow ptosis (66). This move towards image-guided, individualized planning directly parallels the goals of digital surgical simulation, aiming to replace generalized templates with patient-specific maps. Although surgical robotics for hair extraction and implantation are not discussed in the context of eyebrow reconstruction within these references, the drive for enhanced precision, stability, and reduced operator fatigue is a consistent theme in modern procedural refinements. The exploration of new flap designs and suturing techniques for eyebrow and forehead defects, such as modified O-to-T plasties or periosteal tacking sutures, reflects a continuous effort to improve surgical accuracy and reproducibility (67, 68). Furthermore, the critical role of aesthetic planning is emphasized in research analyzing the impact of eyebrow position on perceived character traits. A large rating-based analysis found that eyebrow height significantly influences perceptions of attractiveness, femininity, masculinity, trustworthiness, and dominance (69). Such findings underscore the necessity of achieving a surgical plan that aligns with the patient's aesthetic goals and societal perceptions, a process that would be greatly enhanced by digital tools allowing for virtual eyebrow design and collaborative visualization with the patient to ensure consensus before surgery.

## 7 Future outlook: technological integration and innovation directions

### 7.1 Development of next-generation hair transplantation technologies

The pursuit of next-generation hair transplantation technologies is fundamentally aimed at enhancing precision, consistency, and accessibility, with a particular focus on the development of more intelligent follicular extraction and implantation devices. Robotic-assisted systems represent a significant leap forward in this domain. The future of eyebrow reconstruction will likely be shaped by technological integration. Robotic-assisted systems for follicular unit extraction and AI-based image analysis for pre-operative planning and outcome assessment hold promise for enhancing precision and standardization, though their specific role in eyebrow reconstruction requires further validation (70–74).

### 7.2 Clinical translation pathways for tissue-engineered eyebrow reconstruction

The clinical translation of tissue-engineered eyebrow reconstruction represents a paradigm shift from redistributive transplantation to *de novo* hair follicle generation, with a future path centered on combining patient-specific cells with advanced biomaterials. A foundational element of this approach is the utilization of autologous stem cells. Hair follicle stem cells (HFSCs), residing in the bulge region, are a prime candidate due to their multipotency and role in fueling the hair follicle growth cycle (75). Research has shown that activating these quiescent stem cells is critical for inducing new hair growth, with studies demonstrating that modulating pathways like Wnt/*β*-catenin or autophagy can promote HFSC activation and subsequent hair regeneration (76, 77). Beyond HFSCs, dermal papilla cells (DPCs), which are mesenchymal in origin, are essential for folliculogenesis. The advent of induced pluripotent stem cell (iPSC) technology offers a revolutionary, potentially unlimited source of autologous folliculogenic cells, including DPCs and epithelial cells, for *de novo* hair follicle formation (78). The envisioned clinical pathway involves harvesting a patient's somatic cells (e.g., from blood or skin), reprogramming them into iPSCs, differentiating them into the necessary hair follicle cell types, and then combining them with a customized, bio-active scaffold. This scaffold would be designed not merely as a passive support but as an instructive matrix that guides the three-dimensional organization, specific orientation, and morphological patterning of the developing “follicular unit sheets” or neo-follicles, perhaps within a bioreactor environment to pre-vascularize and mature the construct before transplantation (75). Parallel to scaffold-based tissue engineering, gene editing technologies like CRISPR-Cas9 present a transformative possibility for the ultimate “customization” of reconstructed eyebrows. This technology could theoretically be used to precisely edit the genes in transplanted cells or grafts to regulate critical attributes such as hair growth cycle duration, shaft diameter, pigmentation (color), and even resistance to androgen-driven miniaturization (78). For instance, modulating genes involved in the anagen-catagen transition could prolong the growth phase of transplanted eyebrow hairs, reducing the frequency of trimming. However, the clinical application of germline or even somatic cell gene editing for aesthetic purposes like eyebrow reconstruction is fraught with profound ethical and safety considerations. These include the risks of off-target mutations, long-term unintended consequences, the potential for enhancement beyond therapeutic norms, and the societal implications of accessible “designer” aesthetics (78). Therefore, the translation of tissue-engineered eyebrows will require a stepwise, evidence-based progression from preclinical models to controlled clinical trials, with rigorous oversight to address these complex ethical and biosafety landscapes while proving the efficacy and safety of combining cell therapy, biomaterials, and potentially, genetic modulation.

### 7.3 Establishment of long-term follow-Up databases and outcome evaluation systems

The advancement of eyebrow reconstruction, as with all surgical fields, is critically dependent on robust, long-term outcome data to guide clinical decision-making and evaluate new technologies. A pressing need exists to establish multicenter, standardized postoperative databases that prospectively track outcomes across different techniques—from traditional flap and FUE methods to emerging regenerative approaches. Such databases should systematically collect and analyze long-term data on key metrics including graft survival rates, temporal changes in aesthetic outcomes (such as hair density, direction, and color match), and the incidence and management of both early and late complications (79). Complications in hair transplantation, though often preventable, can arise from poor planning or technique and range from unsatisfactory growth and unnatural appearance to issues like folliculitis, scarring, and in rare cases, keloid formation at donor sites (80, 81). A standardized database would enable the identification of risk factors and the development of evidence-based guidelines to minimize these adverse events. Complementing the database, there is a parallel need to develop and validate objective, quantitative tools for outcome assessment to reduce the bias inherent in subjective surgeon or patient evaluations. Artificial intelligence (AI) and machine learning-driven image analysis represent a powerful frontier in this regard (82). AI algorithms can be trained to perform precise, reproducible measurements from clinical photographs or trichoscopic images, quantifying parameters such as hair density, caliber, growth angle, and coverage percentage over time. This technology can also assist in preoperative planning by analyzing facial symmetry and designing individualized hairline patterns (83). The integration of AI with long-term clinical databases creates a powerful synergy. The objective metrics generated by AI analysis can populate the database with high-fidelity data, which in turn can be used to train and refine more accurate AI models. This cycle generates high-level evidence that can critically evaluate the comparative effectiveness of different surgical techniques, adjuvant therapies (like platelet-rich plasma or stem cell treatments), and patient-specific factors (84, 85). For instance, such a system could provide definitive evidence on whether robotic FUE yields superior long-term density compared to manual FUE for eyebrow restoration, or whether the addition of autologous fat grafting improves graft survival in scarred recipient beds (86). Ultimately, the establishment of a comprehensive, AI-augmented outcomes registry is not merely an academic exercise but a foundational requirement for elevating the practice of eyebrow reconstruction. It enables a transition from anecdotal experience to data-driven medicine, providing the empirical backbone needed to validate innovations, optimize patient selection and surgical protocols, and deliver truly personalized, evidence-based care.

## 8 Conclusion

The evolution of eyebrow reconstruction has transcended its origins as a mere exercise in tissue coverage, entering a sophisticated era defined by the pursuit of natural aesthetics, minimally invasive approaches, and the integration of regenerative repair principles. This journey reflects a broader paradigm shift in reconstructive surgery, where functional restoration is no longer the sole endpoint; it is now inextricably linked with achieving psychologically and socially acceptable cosmetic outcomes. From an expert perspective, the current landscape is not defined by the supremacy of a single technique but by a nuanced, patient-specific toolkit where traditional, contemporary, and future-oriented modalities each hold distinct, complementary roles.

Traditional flap techniques, including scalp, temporal, and island flaps, remain the cornerstone for managing complex, large-scale, or composite defects, particularly those involving underlying structural loss or post-oncological resection. Their irreplaceable value lies in their proven reliability, robust vascularity, and ability to provide bulk and texture-matched tissue in a single stage. However, their limitations—including donor site morbidity, suboptimal hair directionality, and often a “patch-like” appearance—have historically underscored the gap between reconstruction and true aesthetic restoration. The advent and refinement of Follicular Unit Extraction (FUE) for eyebrow restoration marked a pivotal turn towards minimally invasive precision. By enabling the harvesting and implantation of naturally growing follicular units, FUE has dramatically elevated the standard for aesthetic outcomes in cases of partial or non-scarring alopecia of the eyebrows. It allows for meticulous control over hair angle, density, and direction, mimicking the native brow's delicate architecture. This technique has rightly become mainstream for a significant patient subset, offering high satisfaction rates. Yet, its efficacy is inherently constrained by the availability of a suitable donor area, making it less viable for patients with extensive scalp alopecia or those requiring large-volume reconstruction.

The most profound frontier, and the one that promises a fundamental solution to donor limitation, is regenerative medicine, specifically hair follicle tissue engineering and cell-based therapies. Research into dermal papilla cells, epidermal stem cells, and scaffold-based bioengineering represents the potential for *de novo* hair follicle generation. While still largely confined to laboratory models and early-phase clinical trials, these approaches hold the promise of an unlimited, autologous hair source. The current challenges in this domain are substantial, centering on achieving consistent *in vivo* folliculogenesis, ensuring long-term follicular cycle stability, and establishing scalable, clinically translatable protocols. The gap between promising *in vitro* results and robust, reproducible clinical efficacy remains the primary bottleneck.

Therefore, the central challenge for the field is not choosing one path over another but strategically integrating these diverse perspectives. The future of eyebrow reconstruction lies in a balanced, multi-modal philosophy. This requires a careful analysis of each patient's defect characteristics, donor site status, aesthetic goals, and overall health to create a tailored surgical plan. For instance, a combined approach using a flap for structural framework and subsequent FUE for refining hair density and direction can yield superior results. The trajectory of progress is unequivocally towards deeper interdisciplinary convergence. The integration of digital technology—such as 3D imaging and simulation for pre-operative planning—robotic assistance for enhancing the precision of FUE harvesting and implantation, and advanced biomaterials from bioengineering, will be the key drivers.

In conclusion, eyebrow reconstruction has evolved into a sophisticated field where the choice of technique is dictated by a nuanced, patient-specific assessment of defect etiology and extent. For localized defects, FUE offers a minimally invasive solution with precise aesthetic control. For larger or composite defects, traditional flaps remain the workhorse. Adjunctive treatments like laser and regenerative therapies enhance outcomes in scarred beds. The ultimate goal is the seamless unification of form and function, achieved through a balanced, multi-modal philosophy that integrates the reliability of flaps with the finesse of micro grafting.
